# Midazolam inhibits chondrogenesis via peripheral benzodiazepine receptor in human mesenchymal stem cells

**DOI:** 10.1111/jcmm.13584

**Published:** 2018-03-07

**Authors:** Yung‐Ching Chen, King‐Chuen Wu, Bu‐Miin Huang, Edmund Cheung So, Yang‐Kao Wang

**Affiliations:** ^1^ Department of Cell Biology and Anatomy College of Medicine National Cheng Kung University Tainan Taiwan; ^2^ Department of Nursing Chang Gung University of Science and Technology Chia‐Yi County Taiwan; ^3^ Department of Anesthesiology Chang Gung Memorial Hospital Chiayi County Taiwan; ^4^ Department of Anesthesiology An‐Nan Hospital China Medical University Tainan Taiwan; ^5^ Department of Medicine China Medical University Taichung Taiwan

**Keywords:** chondrogenesis, mesenchymal stem cells, Midazolam, peripheral benzodiazepine receptor

## Abstract

Midazolam, a benzodiazepine derivative, is widely used for sedation and surgery. However, previous studies have demonstrated that Midazolam is associated with increased risks of congenital malformations, such as dwarfism, when used during early pregnancy. Recent studies have also demonstrated that Midazolam suppresses osteogenesis of mesenchymal stem cells (MSCs). Given that hypertrophic chondrocytes can differentiate into osteoblast and osteocytes and contribute to endochondral bone formation, the effect of Midazolam on chondrogenesis remains unclear. In this study, we applied a human MSC line, the KP cell, to serve as an in vitro model to study the effect of Midazolam on chondrogenesis. We first successfully established an in vitro chondrogenic model in a micromass culture or a 2D high‐density culture performed with TGF‐β‐driven chondrogenic induction medium. Treatment of the Midazolam dose‐dependently inhibited chondrogenesis, examined using Alcian blue‐stained glycosaminoglycans and the expression of chondrogenic markers, such as SOX9 and type II collagen. Inhibition of Midazolam by peripheral benzodiazepine receptor (PBR) antagonist PK11195 or small interfering RNA rescued the inhibitory effects of Midazolam on chondrogenesis. In addition, Midazolam suppressed transforming growth factor‐β‐induced Smad3 phosphorylation, and this inhibitory effect could be rescued using PBR antagonist PK11195. This study provides a possible explanation for Midazolam‐induced congenital malformations of the musculoskeletal system through PBR.

## 1 INTRODUCTION

Benzodiazepines are widely used sedative drugs for applying regional anaesthesia.^1^ To obtain more specific functions to treat patients, numerous types of benzodiazepine derivatives have been synthesized. Among them, Midazolam (Dormicum^®^) is a short‐acting benzodiazepine derivative that has been extensively used in surgical procedures, premedication and induction for general anaesthesia. Compared with other types of anaesthetics, pharmacokinetics and the drug mechanism of benzodiazepine are well understood.^2^, ^3^ In addition to sedation, benzodiazepines possess anticonvulsant, anxiolytic amnestic, hypnosis and centrally mediated muscle relaxation properties.^4^, ^5^ They produce a sedation effect primarily through modulation of gamma‐aminobutyric acid (GABA) receptors in the central nervous system^6^, ^7^ to block nerve impulses. GABA binds with GABA_A_, GABA_B_ and GABA_C_ receptors. Among them, the GABAA receptor is of particular significance for psychopharmacology because it contains a variety of binding sites at which behaviour‐modifying drugs act to produce some or all of their effects.^8^ Benzodiazepines bind to benzodiazepine sites on GABA receptors and allosterically modulate the response of the channel upon GABA binding.^9^ The function of benzodiazepines acting on GABAA receptors is to increase the amplitude or decay time of GABA‐mediated inhibitory post‐synaptic potentials, thus increasing the inhibitory tone of GABAergic synapses to reduce the firing of neuron populations.^10^

In addition to the central nervous system, benzodiazepines act on peripheral tissues performed with peripheral benzodiazepine receptor (PBR) or mitochondria translocator proteins.^11^, ^12^ A PBR is distinct in its pharmacological, anatomical, structural and physiological aspects when compared with central benzodiazepine receptors.^12^, ^13^ PBR is an 18 kD protein, localize to the mammalian mitochondria membrane, and are highly conserved among various mammalian species in various types of peripheral tissue.^11^ It has been shown that PBR distributes ubiquitously in most types of tissues, including bone marrow stromal cells.^14^ Putative PBR functions are involved in numerous types of physiological processes, such as steroidogenesis, apoptosis, cell proliferation, regulation of mitochondrial membrane potential, mitochondria respiratory chains, voltage‐dependent calcium channels and microglial activation.^11^, ^12^, ^15^, ^16^, ^17^ PK11195 is a competitive antagonist that specifically binds to PBR^18^ and has been shown to reverse the inhibitory effect of Midazolam on the anxiolytic and antidepressant effects of Midazolam.^19^

Although numerous papers have proved the safety of benzodiazepine and its derivatives, there remain in vitro and in vivo studies indicating the toxic effects that can occur after benzodiazepine treatment.^20^, ^21^ For example, previous study has demonstrated that the incident rate of congenital malformations was 0.23% if the maternal plasma was diagnosed as benzodiazepine positive.^21^ Furthermore, Deck et al summarized the database of all infants born with major congenital malformations to mothers on antiepileptic drugs from Boston Medical Center from the years 2003 to 2010, including cleft lips/palate, cardiac defects and urogenital defects. Results showed that for women on benzodiazepine monotherapy during pregnancy, major congenital malformations were high and extended to 10.6%. Among their infants, the rate for cleft lip/palate was approximately 0.747%.^22^ Recent in vitro and in vivo animal studies have suggested that exposure to clinically relevant general anaesthetics at the peak of brain development could be detrimental to immature mammalian neurons as demonstrated by massive and widespread apoptotic neurodegeneration.^23^ In addition, previous studies exposing young mice to benzodiazepine derivatives, either treated alone or together with other chemicals, have resulted in high birth rates of runts, increased retarded ossification of skull bones and a variety of sternal defects.^24^, ^25^ More importantly, Midazolam has been found to inhibit osteogenesis of human bone marrow‐derived mesenchymal stem cells (hMSCs).^26^ However, the detailed mechanisms for benzodiazepine‐regulated bone/cartilage differentiation that associated with foetal growth retardation remain unknown.

As mentioned above, benzodiazepine and its derivatives are associated with birth defects and cleft lip/palates^27^ and have been linked to osteotoxicity.^26^ Because chondrocytes are the precursors of osteogenesis during musculoskeletal development, we sought to investigate in this study the Midazolam‐regulated chondrogenesis of human MSCs and the molecular mechanisms underlying this process.

## 2 MATERIALS AND METHODS

### 2.1 Experimental system

KP cells, an immortalized human MSC line, were established from primary human MSCs, which were transduced using a retroviral vector LXSN‐16E6E7.^28^, ^29^ KP cells display similar types of cell surface marker profiles as parental primary human MSCs do and retain the stem‐like properties. KP cells can not only renew themselves, but also differentiate into mesenchymal and nonmesenchymal cell lineages without neoplastic transformation under certain conditions.^28^ This type of cell line has been used in studies of stem cell biology and regenerative medicine.^30^ The cell line used in this study was kindly provided by Dr. Shih‐Chieh Hung (China Medical University). We also purchased primary human bone marrow‐derived MSCs from Lonza Walksville Inc., which were maintained in growth medium: DMEM low glucose containing 10% foetal bovine serum (GIBCO with selected lot), 100 units/mL penicillin and 100 μg/mL streptomycin (both from GIBCO) at 37°C with a humidified 5% CO_2_ atmosphere and medium was changed twice a week. Only early passages (passages 3‐6) of primary hMSCs were used in this experiment. To induce chondrogenic differentiation, KP cells or hMSCs were treated with chondrogenic induction medium (DMEM low glucose, 0.1 μmol/L Dexamethasone, 50 μmol/L L‐ascorbic acid‐2‐phosphate, 1× Insulin‐Transferrin‐Selenium, 40 μg/mL L‐proline, 100× L‐glutamine, 5 ng/mL transforming growth factor‐β3).^31^, ^32^ For the pelleted culture, 3 × 10^5^ cells per pellet were placed in a 15‐ml centrifuge tube and centrifuged to produce a pellet. The medium was changed every 3 days. For high‐density cultures, cells were seeded at 3 × 10^4^ cells/cm^2^ overnight and the medium was replaced with chondrogenic induction medium the following day.^31^ The chondrogenic differentiation was accessed after 7 or 14 days of treatment.^31^, ^32^

### 2.2 Alcian blue staining and quantification

Cells in the pellet culture or at high densities were rinsed with PBS and fixed with 4% paraformaldehyde for 10 minutes. After fixation, cells were rinsed twice with PBS and then stained with 1% Alcian blue for 30 minutes and rinsed in PBS until the blue colour was removed from the negative control cells. The amount of Alcian blue‐stained glycosaminoglycan (GAG) was quantified as per Woods et al, with modification, by dissolving the stained GAG in 6 mol/L guanidine hydrochloride overnight at 4°C. The absorbance of dissolved stained GAG was then quantified using a spectrophotometer at OD 620 nm.^33^

### 2.3 Western blot analysis

After treatment, cells were rinsed twice using chilled PBS and then lysed and collected using a radioimmunoprecipitation assay buffer (Thermo) plus protease and phosphatase inhibitor cocktails (Thermo). After sonication, cell lysate was centrifuged at 15 000 × *g* for 30 minutes at 4°C and supernatant was collected in an eppendorf tube and stored at −80°C. Protein concentration was assessed using a bicinchoninic acid protein assay (Bio‐Rad) as per the manufacturer's instructions. Fifteen to thirty μg of protein was resolved using SDS‐PAGE, followed by electro‐transferred onto a methanol‐soaked polyvinylidene fluoride (PVDF) membrane. The PVDF membrane was blocked with 5% milk or 2% bovine serum albumin (for phosphorylated protein) in Tris‐Buffered Saline‐Tween 20 (TBST) and then incubated with primary antibody overnight at 4°C. The membrane was then rinsed with TBST and the immunocomplex on membrane was detected using horse‐radish peroxidase‐conjugated secondary antibody, and the final immunocomplexes were visualized using fluorography with an enhanced chemiluminescence reagent (GE Healthcare Life Sciences).

### 2.4 Immunostaining and immunofluorescence

Cells in micropellet cultures were fixed with 4% paraformaldehyde, washed in PBS and then sectioned and frozen (LEICA CM 1950). The 10‐μm‐thick sections were rinsed twice in PBS, followed by soaking in SuperBlock blocking buffer (Thermo) for 1 hour at room temperature, and then incubated with primary antibody overnight at 4°C. The sections were then rinsed twice with PBS and stained with secondary antibody conjugated with Alexa 594 (Thermo). Images were taken using an Olympus epifluorescence microscope. For high‐density cultures, cells were rinsed twice with PBS and then fixed with 4% paraformaldehyde, soaked in SuperBlock blocking buffer (Thermo) for 1 hour at room temperature and then incubated with primary antibodies overnight at 4°C. The sections were then rinsed twice with PBS and stained with secondary antibody conjugated Alexa 594 (Thermo). Images were taken using an Olympus epifluorescence microscope or confocal microscope (Olympus FV‐1000).

### 2.5 Transfection of small interfere RNA

Small interference RNA (siRNA) (ON‐TARGETplus™ siRNA Smartpool) specifically against human PBR was purchased from GE Dharmacon RNAi Technologies, and the transfection of siRNA (5 nmol/L) was performed using a Lipofectamine 2000 transfection reagent (Thermo Fisher Sci) as per manufacturer instructions. siRNA with a scrambled RNA sequence (siN) served as a transfection control. The protein levels of PBR in MSCs after siRNA transfection were detected using Western blot analysis.

### 2.6 Statistics

All quantified results are shown in mean ± SEM of three to four independent experiments. Statistical analyses were performed using ANOVA followed by Tukey's test for significant difference. Significance was accepted when *P* < .05.

## 3 RESULTS

### 3.1 Establishment of chondrogenic differentiation

**Figure 1:**
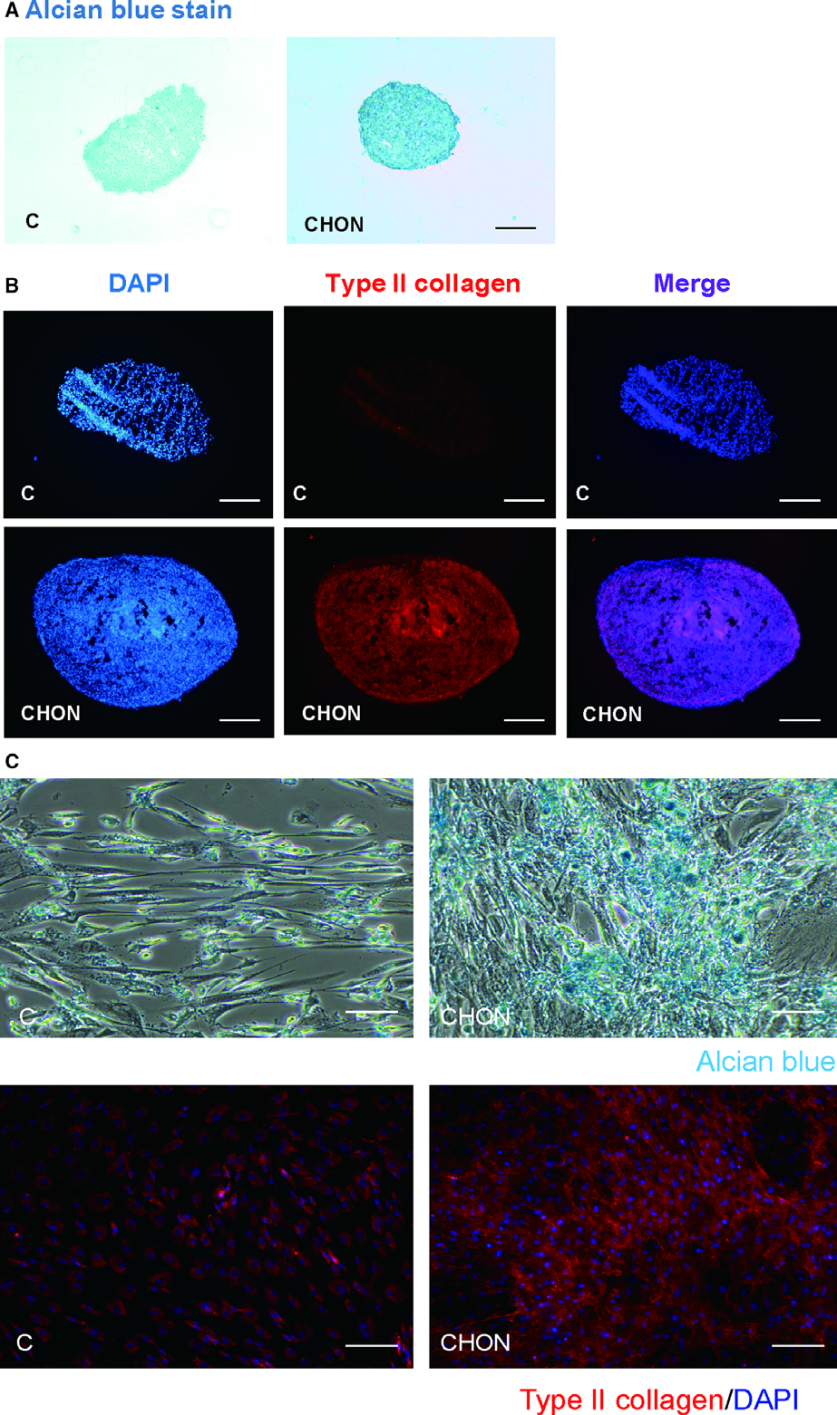
Establishment of chondrogenesis by micropellet and high‐density culture of mesenchymal stem cells. The KP cells were trypsinized and cell number was counted. About 3 × 10^5^ cells were used and centrifuged at 150 × *g* to make a micropellet. For chondrogenic induction, the micropellets were treated with or without chondrogenic induction medium for 14 d. The chondrogenic differentiation was accessed using (A) Alcian blue staining and (B) type II collagen immunofluorescence staining. (C) Chondrogenic differentiation of KP cells at high‐density culture (3 × 10^4^ cells/cm^2^) for 14 d in the presence or absence of chondrogenic induction medium. Chondrogenesis was assessed using Alcian blue staining and immunofluorescence staining of type II collagen. Scale bar: (A) 400 μm, (B, C) 200 μm. The nuclei were stained with DAPI

We first established an in vitro chondrogenic model using either micropellet or high cell density cultures. KP cells were treated with or without transforming growth factor‐β3 (TGF‐β) containing chondrogenic induction medium for 14 days; the chondrogenic differentiation was examined using Alcian blue staining to stain GAGs or by immunostaining of type II collagen. Results demonstrated that without chondrogenic induction, the morphology of the micropellets showed incomplete structures and fractures in the centre of pellets (C), whereas pellets treated with chondrogenic induction medium showed a condensed morphology (CHON) (Figure 1A). In control pellets, cells were lightly stained using Alcian blue, whereas chondrogenic induction medium‐treated micropellets (CHON) were strongly stained (Figure 1A). In addition to the expression of GAG, we also examined the expression of type II collagen in micropellets performed with immunostaining. The results showed that the staining of type II collagen was not clear in control cells, but chondrogenic induction medium‐treated micropellets showed high staining of type II collagen (Figure 1B). Similar results could also be found in cells seeded at a high seeding density (3 × 10^4^ cells/cm^2^) where Alcian blue and type II collagen were able to be stained in chondrogenic induction medium‐treated cells (CHON) (Figure 1C), but not in control cells (C). These results suggest that in vitro chondrogenic differentiation was successfully established and can be used in further studies.

### 3.2 Midazolam inhibits chondrogenic differentiation

**Figure 2:**
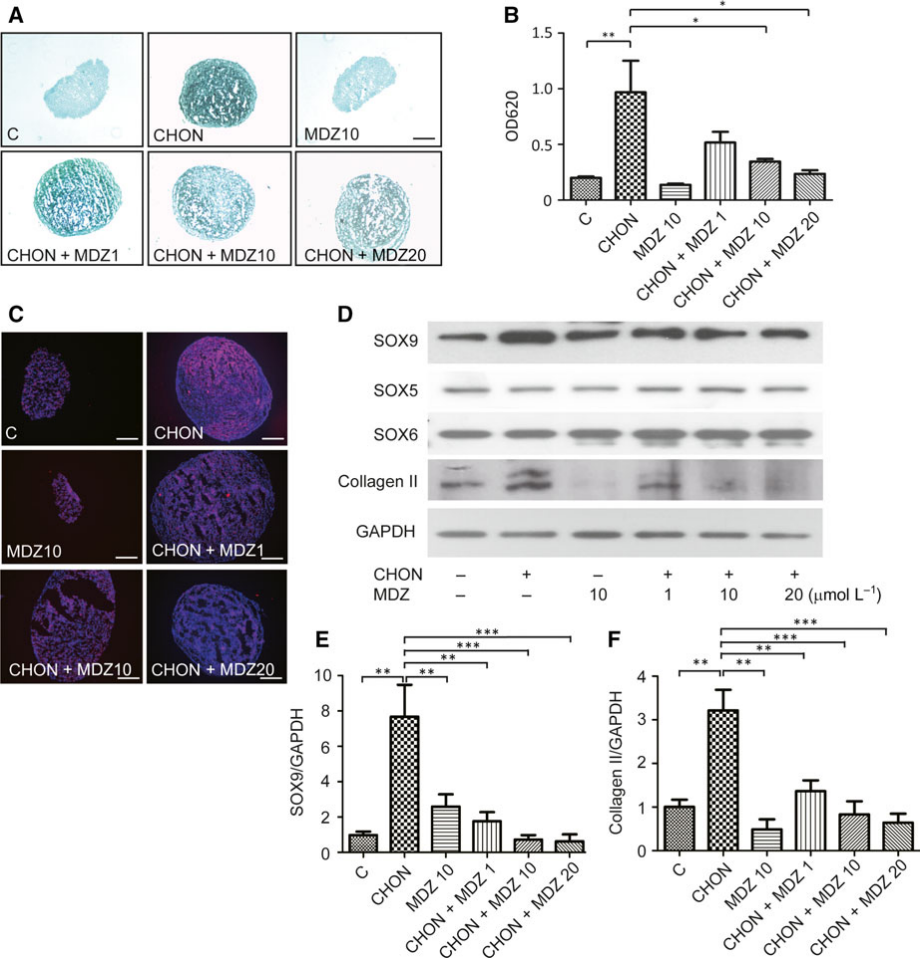
Midazolam dose‐dependently inhibits chondrogenic differentiation. KP cells in micropellets were treated without (C) or with chondrogenic induction medium (CHON) and in the presence of various concentrations of Midazolam (1, 10 and 20 μmol/L, denoted as CHON + MDZ 1, CHON + MDZ 10 and CHON + MDZ 20, respectively) for 14 d. Midazolam only at concentration of 10 μmol/L (MDZ10) was used as a negative control. Control pellets (C) were treated with serum‐free medium plus insulin‐transferrin‐selenium. Chondrogenesis was evaluated using (A, B) Alcian blue staining and (C) immunofluorescence staining of type II collagen. (B) Alcian blue was quantified by dissolving it in guanidine hydrochloride, after which the optical density of the samples was read by a spectrophotometer at OD620 nm. Quantification results are normalized with untreated control. Scale bar: (A) 400 μm; (C) 200 μm. (D) KP cells were seeded at a high density (3 × 10^4^ cells/cm^2^) and treated with or without chondrogenic induction medium in the presence of various doses of Midazolam for 7 d. Protein levels of chondrogenic marker SOX5, SOX6, SOX9 and type II collagen were examined using Western blot analysis. The protein level of GAPDH served as an internal control. Quantification of SOX9 (D) and type II collagen (E) results are presented as mean ± SEM of three independent experiments (**P ≦ .01, ***P ≦ .005)

To test for Midazolam‐inhibited hMSC chondrogenesis, KP cells either in micropellets or in high‐density cultures were treated without (C) or with TGF‐β‐containing chondrogenic induction medium (CHON) in the presence of different doses of Midazolam (1, 10 and 20 μmol/L, denoted as CHON + MDZ1, CHON + MDZ10 and CHON + MDZ20, respectively). The Midazolam only group at dose of 10 μmol/L (MDZ10) was used as a negative control. The chondrogenic differentiation was analysed using Alcian blue, immunostaining of type II collagen as previously described. TGF‐β‐containing chondrogenic induction medium significantly increased Alcian blue content in micropellet cultures, and this chondrogenic induction was dose‐dependently inhibited by the treatment of Midazolam (Figure 2A,B). Similar results from the immunostaining of type II collagen can be observed in Figure 2C. At high cell density cultures, the chondrogenic differentiation was inhibited by Midazolam dose‐dependently (Figure S1). Early studies have indicated that SOX trios (SOX5, SOX6 and SOX9) are crucial for chondrogenesis.^34^, ^35^ To further characterize Midazolam‐inhibited protein levels of chondrogenic markers, we performed Western blot analysis to examine the protein levels of SOX5, SOX6, SOX9 and type II collagen. KP cells were seeded at density of 3 × 10^4^ cells/cm^2^ and were treated with or without chondrogenic differentiation medium in the presence of various doses of Midazolam (1, 10 and 20 μmol/L) for 7 days. The chondrogenic induction successfully increased the protein levels of SOX9 and type II collagen, whereas Midazolam significantly inhibited protein levels of SOX9 and type II collagen (Figure 2D‐F). Interestingly, the chondrogenic induction did not affect the protein levels of SOX5/SOX6 and treatment of Midazolam did not alter the protein levels SOX5/SOX6. These results demonstrate that Midazolam‐inhibited chondrogenic differentiation is mediated by the inhibition of the expression of chondrogenic‐associated proteins.

### 3.3 Midazolam inhibits chondrogenic differentiation of primary mesenchymal stem cells

**Figure 3:**
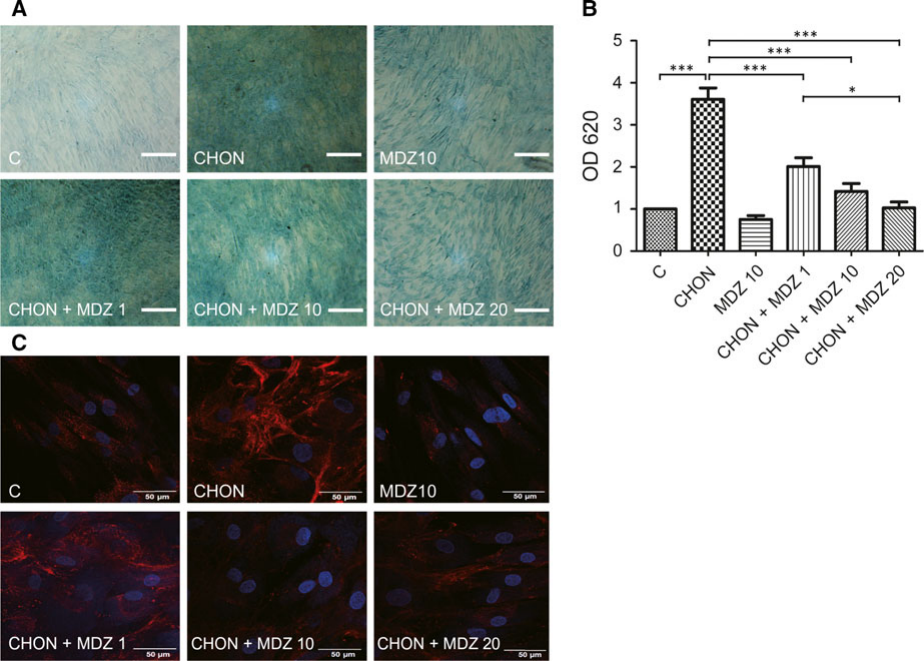
Midazolam inhibits chondrogenic differentiation in primary mesenchymal stem cells. Primary mesenchymal stem cells were seeded at 3 × 10^4^ cells/cm^2^ and treated without (C) or with chondrogenic induction medium (CHON) in the presence of various concentrations of Midazolam (1, 10 and 20 μmol/L, denoted as CHON+ CHON + MDZ 1, CHON + MDZ 10 and CHON + MDZ 20, respectively) for 7 d. Midazolam only at concentration of 10 μmol/L (MDZ10) was used as a negative control. Chondrogenesis was evaluated using (A) Alcian blue staining and (B) Alcian blue was quantified by dissolving it in guanidine hydrochloride, after which the optical density of the samples was read by a spectrophotometer at OD620 nm. Quantification results are normalized with untreated control. Results are presented as mean ± SEM of three independent experiments (*P ≦ .05, **P ≦ .01, ***P ≦ .005). (C) Immunostaining of type II collagen. Scale bar: (A) 200 μm; (C) 50 μm

Given that KP cells have been transfected using exogenous genes to maintain MSC stemness,^28^, ^29^ whether the incorporated genes produce different responses in these cells remain unclear. To test the inhibitory effects of Midazolam in chondrogenesis, we performed primary hMSCs to evaluate the results that were obtained from the KP MSC cell line. Primary hMSCs were seeded at a high density (3 × 10^4^ cells/cm^2^) with or without chondrogenic induction medium in the presence of Midazolam (0, 1, 10 and 20 μmol/L) at the indicated time, and chondrogenic differentiation was evaluated using Alcian blue staining and immunostaining of type II collagen. Similar to the results that were obtained from KP MSCs, chondrogenic induction medium successfully induced an increase in Alcian blue staining, whereas Midazolam inhibited Alcian blue staining dose‐dependently (Figure 3A,B). In addition, chondrogenic induction‐induced type II collagen was dose‐dependently inhibited by the treatment of Midazolam (Figure 3C). Similar was confirmed using micropellet culture of primary hMSCs (Figure S2). Together, these results demonstrate that Midazolam‐inhibited chondrogenic differentiation is reproducible in MSCs from different sources.

**Figure 4:**
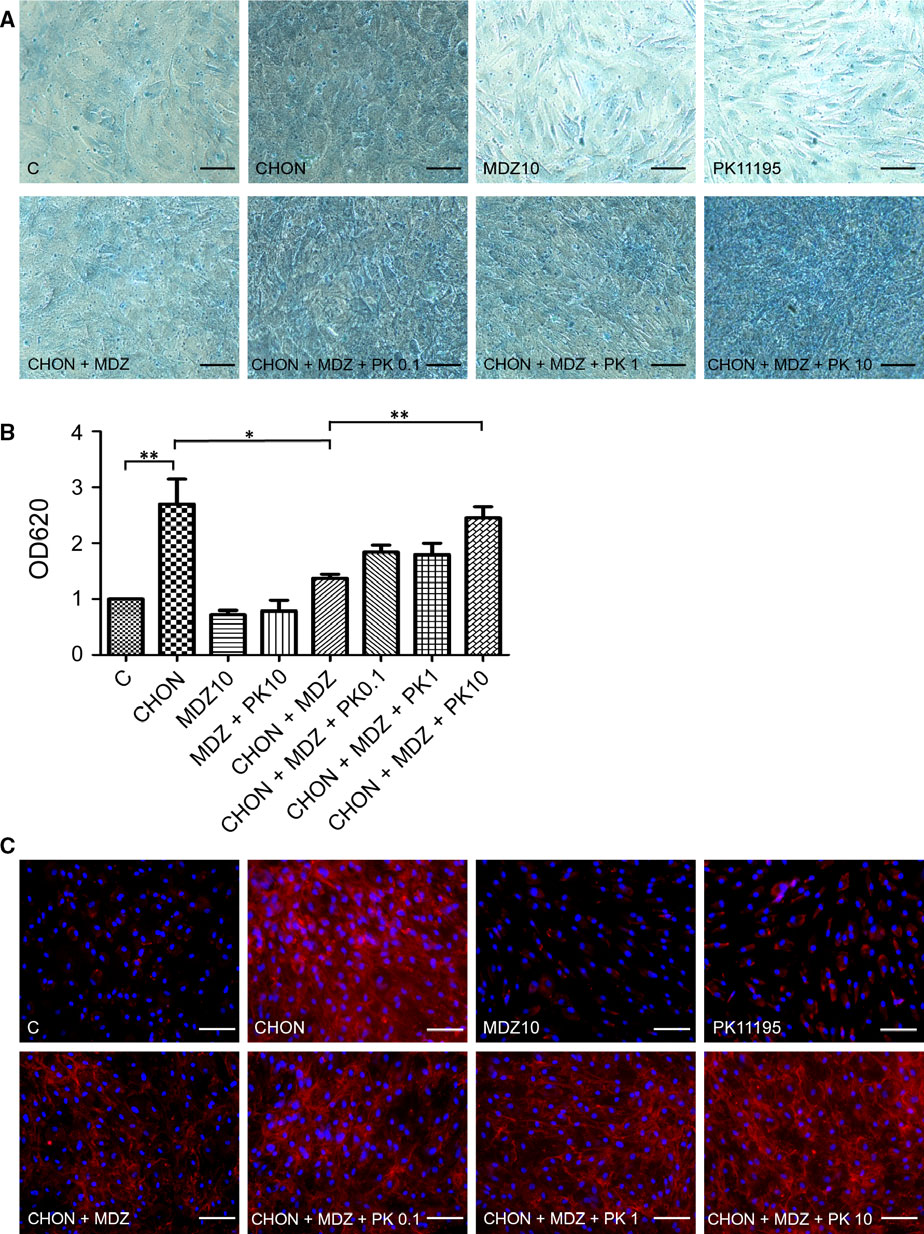
Peripheral benzodiazepine receptor antagonist PK11195 rescues Midazolam‐induced suppression of chondrogenesis. Primary mesenchymal stem cells were seeded at 3 × 10^4^ cells/cm^2^ and treated without or with chondrogenic induction medium in the presence of Midazolam (10 μmol/L, MDZ10, CHON + MDZ, respectively) or co‐treated with PK11195 (0.1, 1 and 10 μmol/L, denoted as PK0.1, PK1 and PK10, respectively) for 7 d. Cells were stained with Alcian blue (A, B) or immunoblotted with type II collagen (C). (B) Quantification of Alcian blue and these quantification results are normalized with untreated control. Representative images are selected from at least three independent experiments. Results are presented as mean ± SEM of three independent experiments (*P ≦ .05, **P ≦ .01). Scale bar: 50 μm

Because the cell type that we used was taken from peripheral tissue, we sought to understand whether Midazolam‐inhibited chondrogenesis was mediated by PBR. We first examined PBR inhibitor PK11195 on the effect of Midazolam on chondrogenesis. Primary hMSCs were seeded at a high density (3 × 10^4^ cells/cm^2^) with or without chondrogenic induction medium in the presence of Midazolam (10 μmol/L) and co‐treated with PK11195 (0, 0.1, 1 and 10 μmol/L) for 7 days. The chondrogenic differentiation was accessed using Alcian blue staining and immunostaining of type II collagen. We found that treatment of PK11195 using a 10 μmol/L dose significantly improved the Midazolam‐inhibited effect on chondrogenic differentiation (Figure 4A,B). Similar results were obtained using immunostaining with type II collagen (Figure 4C).

**Figure 5:**
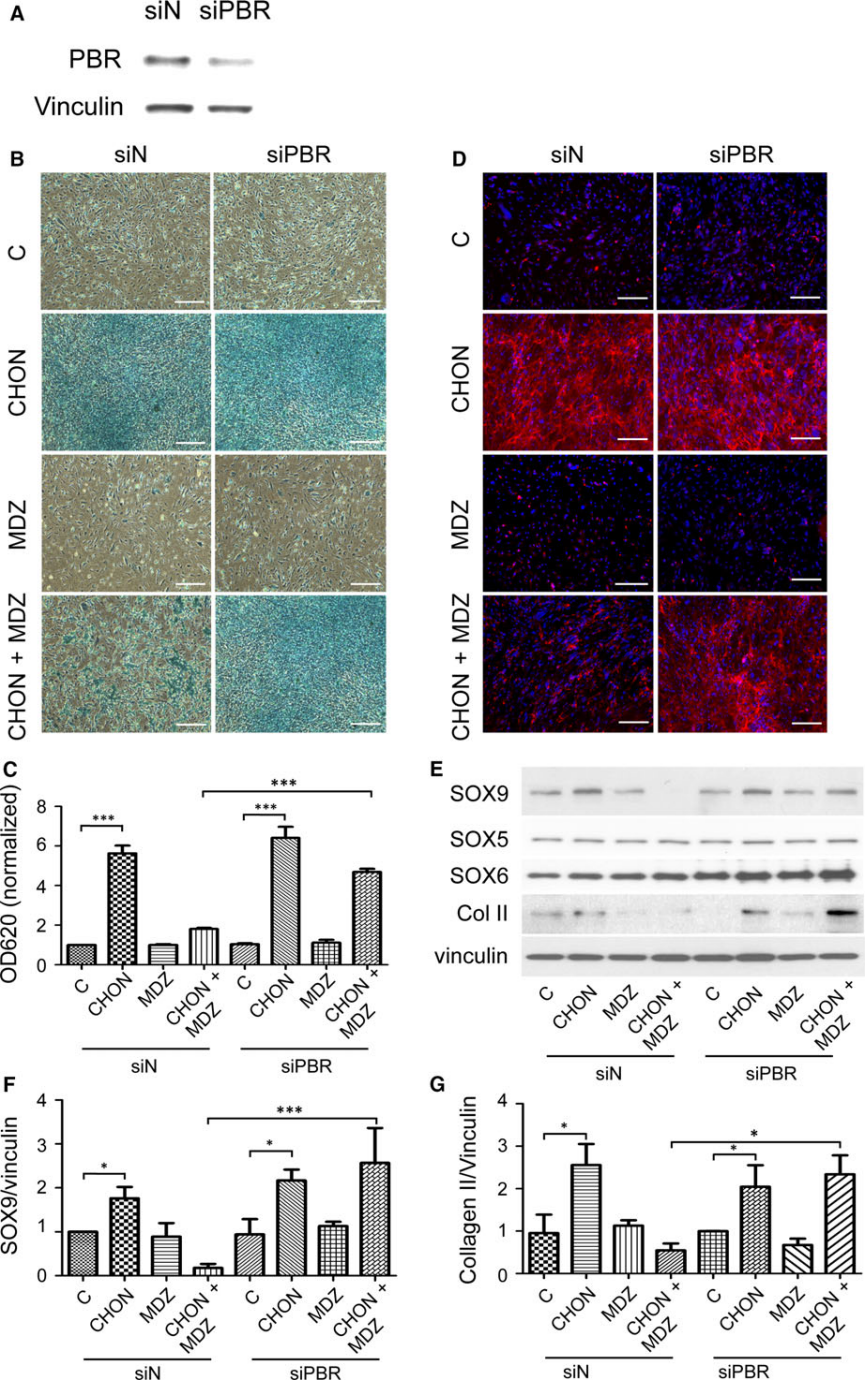
Knockdown of peripheral benzodiazepine receptor (PBR) by siRNA rescues Midazolam‐induced suppression of chondrogenesis. The specific small interference RNA against human PBR (siPBR) was transfected to knockdown PBR expression in primary mesenchymal stem cells (MSCs). A scrambled siRNA (siN) served as a negative control. (A) The protein level of PBRs in transfected cells was accessed using Western blot analysis. Cells were then treated without or with chondrogenic induction medium in the absence or presence of Midazolam (10 μmol/L, MDZ, CHON + MDZ, respectively) for 7 d. Cells were then fixed and stained with (B, C) Alcian blue or (D) immunostained with type II collagen. The nuclei were stained with DAPI. C, Quantification of Alcian blue and these quantification results are normalized with untreated control. (E‐G) Primary MSCs, transfected with either siN or siPBR, were seeded at high cell density and then treated with or without chondrogenic induction medium or co‐treated with or without Midazolam (10 μmol/L) for 7 d. Protein levels of SOX5, SOX6, SOX9 and type II collagen were analysed using Western blot analysis. Levels of vinculin served as an internal control (upper panel). Quantification results of (F) SOX9 and (G) type II collagen are shown. Quantification results are presented as mean ± SEM of three independent experiments (*P ≦ .05, ***P ≦ .005)

Our results indicate that treatment of cells with a higher dose of PK11195 (20 μmol/L) resulted in a decrease in cell number, which represented an off‐target effect of PK11195 (data not shown). We therefore performed siRNA specific to PBR to elucidate the effect of PBR in our model. The control (siRNA with a scrambled sequence, siN, 5 nmol/L) or siPBR (5 nmol/L) was transfected into primary hMSCs. Protein levels of PBR after siRNA transfection were determined using Western blot analysis, and the results showed the successful knockdown by siPBR of the endogenous PBR protein levels (Figure 5A) without altering cell numbers (data not shown). The transfected primary MSCs were then seeded at a high density (3 × 10^4^ cells/cm^2^) in the presence of chondrogenic medium with or without Midazolam (10 μmol/L) for 7 days. The chondrogenic differentiation was determined using Alcian blue staining (Figure 5B,C) and immunostaining of type II collagen (Figure 5D). Transfection of scrambled siRNA did not rescue Midazolam‐suppressed Alcian blue and type II collagen content during chondrogenesis. In addition, knockdown of PBR (siPBR) rescued the Midazolam‐inhibited chondrogenic differentiation (Figure 5). Similar results were found using Western blot to detect the protein levels of SOX9 and type II collagen (Figure 5E‐G). However, knockdown of PBR did not affect the protein levels of SOX5/SOX6. These results suggest that inhibition of PBR by either PK11195 or siRNA rescues Midazolam‐suppressed chondrogenic differentiation, which indicates that PBR mediates Midazolam‐inhibited chondrogenic differentiation.

**Figure 6:**
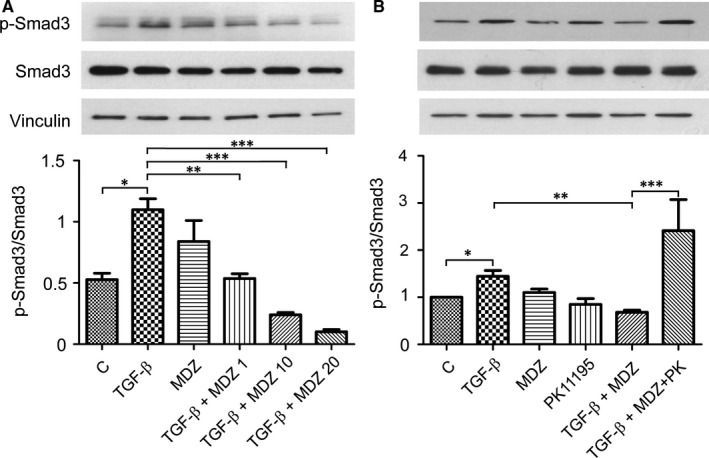
Midazolam inhibits transforming growth factor (TGF)‐β‐induced Smad3 phosphorylation. KP cells were seeded at 3 × 10^4^ cells/cm^2^, serum starved for 16 h and then treated with TGF‐β (5 ng/mL) in the presence of various doses of Midazolam (0, 1, 10 and 20 μmol/L) for 1 h. (A) Levels of Smad3 phosphorylation; total Smad3 and vinculin were analysed using Western blot analysis. Quantification results are presented as mean ± SEM of three independent experiments (*P ≦ .05, **P ≦ .01, ***P ≦ .005). (B) KP cells were seeded at 3 × 10^4^ cells/cm^2^, serum starved for 16 h and then treated with TGF‐β (5 ng/mL) in the presence of Midazolam (10 μmol/L, MDZ) or co‐treated with PK11195 (10 μmol/L, PK). Levels of Smad3 phosphorylation; total Smad3 and vinculin were analysed using Western blot analysis. Quantification results are presented as mean ± SEM of three independent experiments (*P ≦ .05, **P ≦ .01, ***P ≦ .005)

To further characterize that Midazolam inhibits chondrogenesis via inhibition of TGF‐β signalling, we assessed the phosphorylation of Smad3, one of the major downstream signalling molecules of TGF‐β.^36^ We found that administration of Midazolam significantly suppressed TGF‐β‐induced Smad3 phosphorylation dose‐dependently (Figure 6A). Interestingly, if cells were co‐treated with PBR antagonist PK11195, the Midazolam‐induced suppression of Smad3 phosphorylation could be rescued (Figure 6B).

In summary, TGF‐β induces Smad3 phosphorylation. Phosphorylated Smad2/3 can interact with Smad4 and translocate into the nucleus to trigger chondrogenic‐related gene transcription. Midazolam‐induced suppression of chondrogenesis may be mediated by the interaction with PBR, which in turn ameliorates TGF‐β‐induced Smad signalling.

## 4 DISCUSSION

The present study demonstrates the role of Midazolam in chondrogenesis inhibition. However, other recent studies have demonstrated that chondrocytes are the precursors of osteogenesis,^37^, ^38^ but because they have only demonstrated the possible osteotoxicity of benzodiazepine,^26^ the role of Midazolam in chondrogenic differentiation remains unclear. We were interested in whether Midazolam‐induced congenital musculoskeletal defects were caused by the inhibition of chondrogenic differentiation. We first demonstrated benzodiazepine derivative Midazolam‐inhibited chondrogenic differentiation via PBR in human MSCs. Inhibition of PBR by either PK11195 or siRNA specific to PBR rescued the inhibitory effect of Midazolam. More importantly, Midazolam‐inhibited TGF‐β‐induced SMAD3 phosphorylation was mediated by PBRs. The results provided here indicate that PBR plays a crucial role in Midazolam‐inhibited chondrogenesis, which may correlate with congenital malformation in pregnant women who have been treated with benzodiazepine for anxiety or insomnia.

Although benzodiazepine derivatives may cause musculoskeletal defects, the molecular mechanisms of how these benzodiazepine derivatives result in congenital defects remain unknown. To confirm the inhibitory effect of Midazolam, we applied TGF‐β‐containing chondrogenic induction medium^31^, ^32^ in human MSCs. We used several differentiation markers to indicate chondrogenesis, including SOX9, type II collagen and an increased amount of GAGs, which could be stained using Alcian blue. SOX9 is a transcriptional factor that plays a key role in the regulation of chondrogenesis, and multiple signalling pathways are known to regulate the expression and activity of SOX9 during chondrogenesis^39^, ^40^, ^41^, ^42^. Activated SOX9 in turn regulates chondrogenic‐dependent markers, such as type II collagen^43^, ^44^ and aggrecan.^45^ In addition, SOX9, together with SOX5 and SOX6 to form SOX trios, and expression of these trios lead to chondrogenesis.^34^, ^35^ In current study, we found that Midazolam inhibits protein levels of SOX9 but not SOX5 and SOX6 in chondrogenic induction. One possible reason is that SOX9 is found to be one of the TGF‐β/Smad3 downstream effectors^46^ to regulate chondrogenesis. The down‐regulation of SOX9 may result in disruption of the formation of SOX trio, which in turn inhibits the transcription of chondrogenesis‐related genes. On the other hand, we have discovered that Midazolam suppressed chondrogenic differentiation markers at the range of 1‐20 μmol/L in human MSCs. Interestingly, the plasma concentrations of benzodiazepine derivatives from clinical samples ranged from 0.1 to 50 μmol/L.^47^, ^48^ These results suggest that administration of Midazolam at clinical dosages suppresses chondrogenic differentiation by inhibiting the levels of chondrogenic‐dependent markers.

We also investigated how Midazolam acts on MSCs to suppress their chondrogenic differentiation. Because MSCs were isolated from peripheral tissues, we aimed for the PBR. Early reports showed that PBR is expressed ubiquitously in most types of tissues, including bone marrow^14^ and is required for adipogenic differentiation and MSC proliferation.^49^ It is also known that PBR expresses in murine osteoblasts and osteoclasts, suggesting that PBR may be involved in bone homeostasis.^50^ To confirm that PBR mediated inhibitory effects of Midazolam, we used a specific antagonist, PK11195, which has been widely used to study PBR functions.^18^, ^51^ We demonstrated that Midazolam‐induced suppression of chondrogenesis was blocked by PK11195 as shown by chondrogenic markers, such as SOX9 and type II collagen, and by the expression of GAG. This suggests that Midazolam has an inhibitory function against MSCs differentiating into chondrocytes through PBR. However, several studies have indicated that certain types of GABA receptor subunits can be detected in murine MSCs, chondrogenic cells, growth plate chondrocytes, cartilage and osteoblasts.^52^, ^53^, ^54^ It is uncertain whether these subunits are able to assemble to become a functional GABA receptor and whether the GABA receptor can be expressed in hMSCs, which in turn mediate the effect of Midazolam on chondrogenesis. Together, these results suggest that Midazolam acts on hMSCs through PBRs to inhibit chondrogenesis.

At higher doses of PK11195 treatment (higher than 20 μmol/L), we observed cell loss (data not shown), which may have been damaging to our conclusion. To rule out this possible off‐target effect of PK11195, we used siRNA specific to PBRs to evaluate the function of PBR on the actions of Midazolam in chondrogenesis. Twenty‐four hours after transfection of siRNA, the protein level of PBRs was significantly decreased compared with the scrambled control. Midazolam‐induced suppression of chondrogenesis was markedly rescued with siRNA‐down‐regulated PBR in hMSCs. This result further confirmed the role of PBR in mediating the inhibitory effect of Midazolam in chondrogenesis.

The major signalling pathway used to induce chondrogenesis in this study was TGF‐β signalling. TGF‐β, a pleiotropic cytokine, is involved in the regulation of cell division and suppression of immune response.^55^ Upon binding of TGF‐β to its receptor, the receptor complex recruits and phosphorylates the carboxy terminus of receptor‐regulated Smad proteins (R‐Smads: Smad2 and Smad3).^36^, ^56^ Phosphorylated Smads can interact with Smad4 to become a complex, and this complex translocates into the nucleus and turns on gene transcription.^55^ Smad3 is able to activate SOX9 via recruitment of CREB‐binding protein/p300.^46^ In addition, TGF‐β/Smad signalling is essential for osteocartilage development, bone homeostasis and MSC fate decision.^57^ Disruption of TGF‐β/Smad signalling in vivo resulted in serious problems in the musculoskeletal system.^58^ Midazolam has been found to inhibit protein phosphorylation. For example, a previous report indicated that Midazolam inhibits platelet activation by inhibiting protein phosphorylation of protein kinase C.^59^ In mouse hippocampal slices, Midazolam was found to inhibit extracellular signal‐related kinase 1/2 activity, and this inhibition was likely mediated via N‐methyl‐d‐aspartate receptors, phospholipase C and PKC‐dependent signalling.^60^ We therefore proposed that the chondrogenic inhibitory effect of Midazolam was caused by the inhibition of Smad 3 phosphorylation. Indeed, Midazolam can dose‐dependently inhibit Smad3 phosphorylation (Figure 6) and this Smad3 phosphorylation inhibition was mediated by PBR, as proved when administration of PK11195 reversed the inhibitory effect of Midazolam. We therefore speculate that the inhibitory effect of Midazolam on TGF‐β‐induced Smad 3 phosphorylation is mediated by binding to PBR. Several studies have demonstrated that in most types of tissue, PBR is primarily located in the outer membrane of mitochondria.^13^ In addition to the outer mitochondrial membrane, PBR was also located in various subcellular regions. Mukherjee and Das first reported that although the amount of [^3^H]‐labelled R05‐4864 (another PBR ligand) binding sites was highest in the mitochondrial fraction, the nuclear and cytosolic fractions also contained significant amounts of these binding sites.^61^ A study conducted by Kuhlmann and Guilarte discovered that after neural injury, PBR localization was in cytosol or perinuclear areas in microglia cells and macrophages.^62^ In addition, subcellular localization of PBR was also found in cytosol or in the nuclei of different cell types (ie MA‐10, MCF‐7 and MDA‐MB‐231), and these nuclear localized PBR was implicated to correlate with cell proliferation and nuclear cholesterol transport.^63^ This cytosolic localization of PBR supports our speculation that Midazolam can bind with cytosolic PBR to regulate TGF‐β‐induced Smad3 phosphorylation, which also occurs in cytosol. Further study of how Midazolam binds to cytosolic PBRs and exerts its function should be investigated.

Taken together, our findings suggest that Midazolam inhibits chondrogenesis via PBR to inhibit TGF‐β signalling, which in turn suppresses chondrogenesis. This partially explains why this sedative drug can result in congenital malformation of newborns if a pregnant woman is treated with benzodiazepine for anxiety and insomnia. Thus, the future application of these types of drugs must be more thoroughly considered. Further studies to elucidate how these benzodiazepine derivatives interact with their receptors and cause various outcomes require more sophisticated investigation.

## CONFLICT OF INTEREST

The authors declare that there is no conflict of interest.

## AUTHOR CONTRIBUTIONS

YCC, KCW, ECS and YKW initiated the project; YCC and KCW designed the experiments and YCC conducted experiments of the project; BMH provided reagents; YCC, KCW, ECS and YKW analysed the results; YCC, KCW, ECS and YKW wrote manuscript; BMH edited the manuscript; YKW and ECS supervised the progress. All authors read and confirmed the final draft of the manuscript.
