# Supplementary figures and images for: Midazolam inhibits chondrogenesis via peripheral benzodiazepine receptor in human mesenchymal stem cells
Source: J Cell Mol Med. 2018 Mar 7;22(5):2896–907. doi: 10.1111/jcmm.13584 (PMC5908119; doi:10.1111/jcmm.13584)

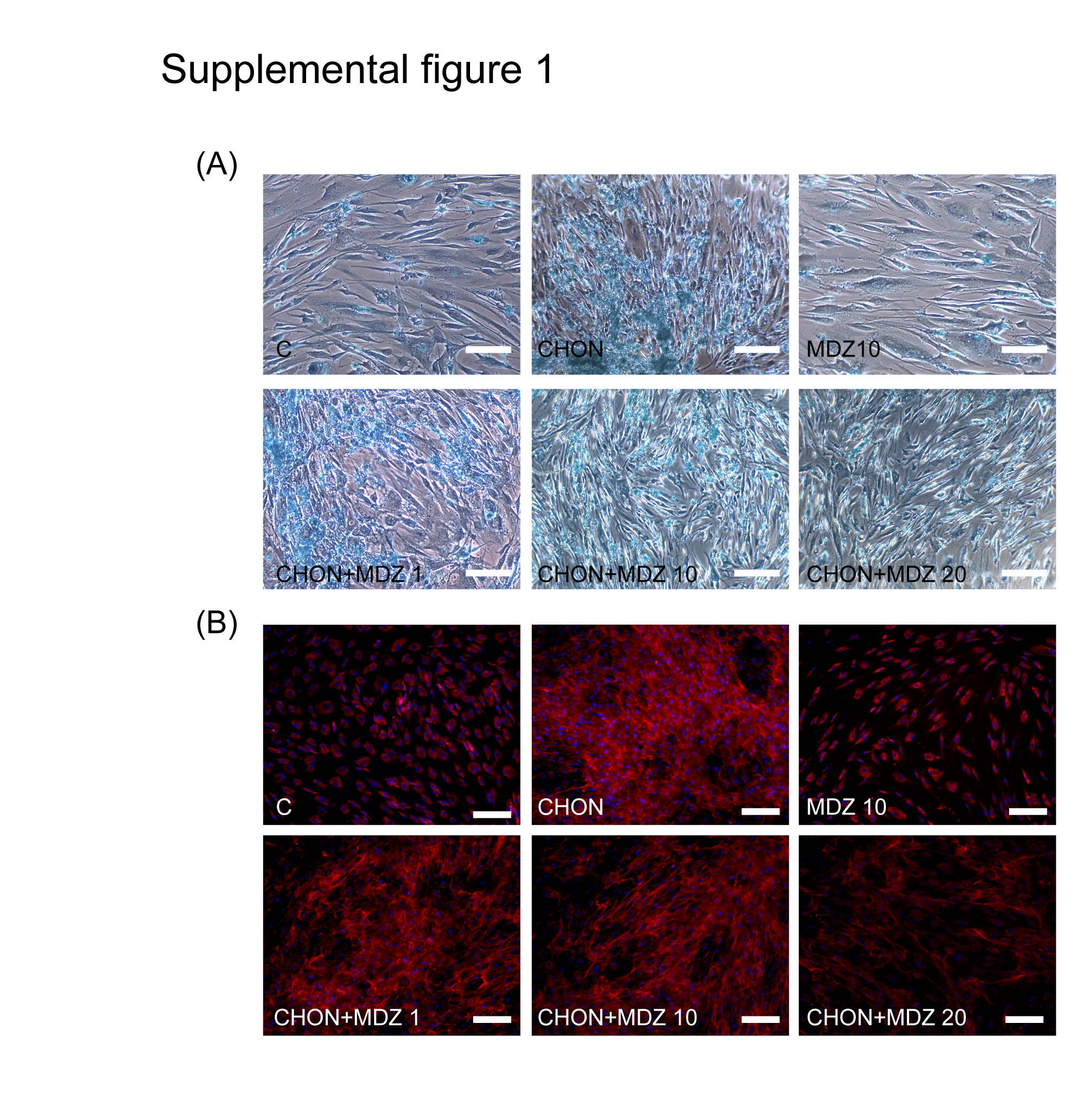

Supplement: Supplementary file 1 [file JCMM-22-2896-s001.tif]

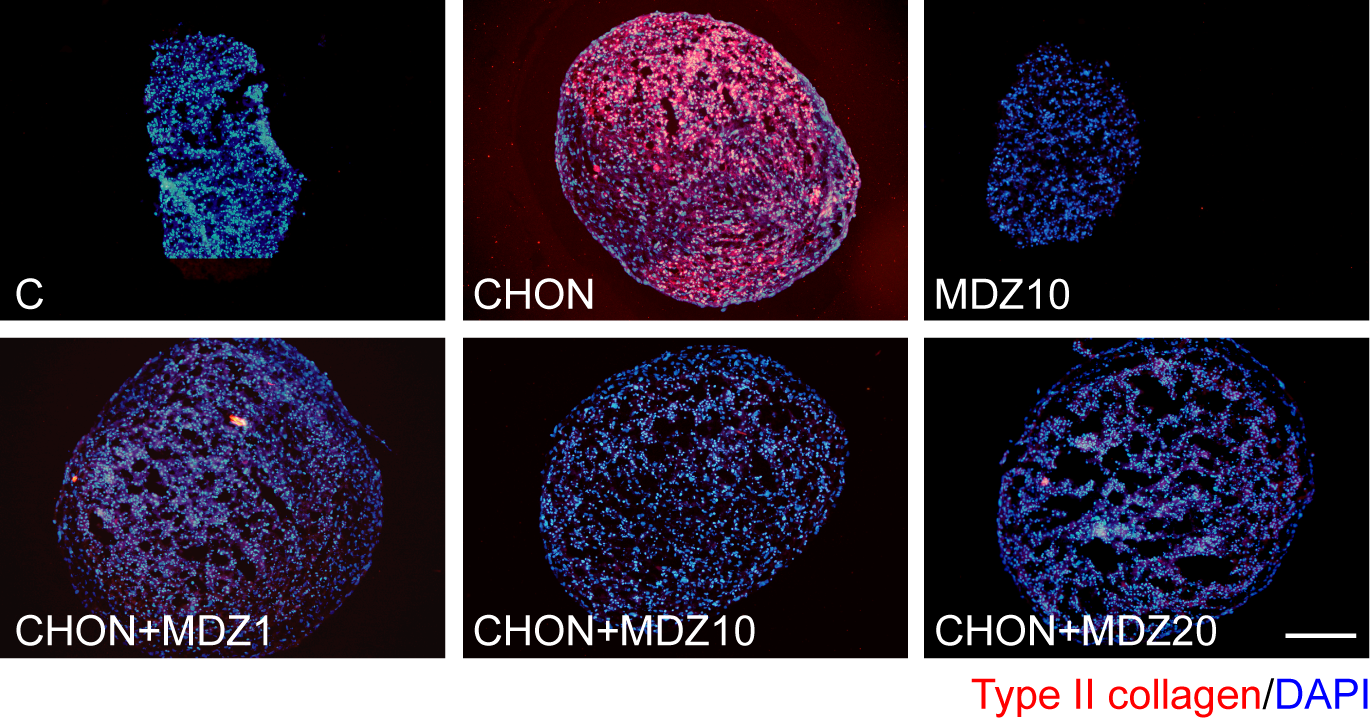

Supplement: Supplementary file 2 [file JCMM-22-2896-s002.tif]
