# Supplementary material for: Midazolam inhibits chondrogenesis via peripheral benzodiazepine receptor in human mesenchymal stem cells
Source: J Cell Mol Med. 2018 Mar 7;22(5):2896–907. doi: 10.1111/jcmm.13584 (PMC5908119; doi:10.1111/jcmm.13584)
Supplement: Supplementary file 3 [file JCMM-22-2896-s003.docx]

Supplemental file

Figure 1. KP cells were seeded at the density of 3x10^4^ cell/cm^2^ and then treated without (C) or with chondrogenic induction medium in the presence of Midazolam (1, 10, 20 μM, denoted as CHON+MDZ1, CHON+MDZ10, and CHON+MDZ20, respectively) for 14 days. Midazolam only at dose of 10 μM (MDZ10) was used as a negative control. Cells were then fixed and stained with (A) Alcian blue (B) immunostaining of type II collagen. Scale bar: (A) 50μm, (B) 200 μm. The nuclei were stained with DAPI.

Figure 2. Primary hMSCs in micropellets were treated without (C) or with chondrogenic induction medium (CHON) and in the presence of various concentrations of Midazolam (1, 10, and 20 µM, denoted as CHON+MDZ 1, CHON+MDZ 10, and CHON+MDZ 20, respectively) for 14 days. Chondrogenesis was evaluated using immunostaining of type II collagen. Scale bar: 400 μm.
